# Revivification

**Published:** 1883-04-20

**Authors:** S. Waterman

**Affiliations:** New York


### REVIVIFICATION.

By S. WATERMAN, M. D., NEW YORK.

The following two cases are selected to show the necessity of making persistent efforts at revivification in cases of sudden death, especially from heart disease, as well as in cases of still-born children.

There is no doubt in my mind that in many cases of sudden death, especially from heart disease, prompt and persistent efforts to reanimate the appearently dead person may result in restoring life. It is probable that in many instances the heart's action may fail from transient causes; a dangerous syncope may supervene, and unless timely efforts are made, and proper measures are promptly resorted to, the person may pass from a state of suspension of vitality into the silent and lasting embrace of death.

A case in point happened to me in the month of February, 1880. Mr. B——, a gentleman of sound constitution, about six feet in height, springing from a healthy ancestry, aged eighty-four years, had an attack of senile gangrene in the inguinal region, two inches and a half in length, and one inch wide. The slough was in time thrown off, and healthy granulation filled the wound. A second attack subsequently, not quite so severe, destroyed a part of the integument in the umbilical region. Its cause and final cure was similar to the first attack. The third attack was in the great toe of the left foot. The entire toe perished, but a line of demarcation formed, the destructive process went no farther, healthy granulation formed, and the healing process progressed in the most healthy and satisfactory manner. Mr. B—— suffered in addition from valvular disease of the heart, and likewise from Bright's disease (granular degeneration), probably in consequence of retarded circulation and diminished blood-pressure. One morning while I was sitting at his bedside, and in friendly conversation with him, he being to all appearance in a very happy mood of mind, he suddenly fell back, his eyes became fixed and glassy, a deadly pallor crept over his countenance, respiration and the heart's action ceased simultaneously, and death seemed to have carried him off suddenly and unexpectedly.

It was this suddenness of the event that impelled me to make efforts at revivification. Two nephews of Mr. B——, who were fortunately in the house, were brought under requisition, and under my direction systematic artificial movements were carried on for nearly thirty minutes. Then, to my unspeakable satisfaction, one deep inspiratory effort was made by the patient himself. Thus encouraged, we redoubled our efforts for ten minutes more; other inspiratory efforts followed in quicker succession, the heart began to respond, hardly audible at first, it required force and momentum; it could now be felt at the wrist; the deadly pallor passed away, the eyes lost their glassy, fixed aspect, sighs and groans could be heard; twitchings of the muscles of the arm and fingers could be distinctly felt, and the rigidity of death made way for re-animated conditions.

He lay unconscious for more than ten hours, respiration being hurried, and breathing stertorous, the heart's action wild and irregular. During the night he was delirious and restless. Toward morning all outward symptoms subsided, and a quiet sleep followed the extreme restlessness.

When I saw him next morning, he sat up in his bed and partook of a good breakfast. Consciousness had returned, and all the life functions were in full operation. He died six weeks afterwards under symptoms of uræmic toxication. During these six weeks, up to the hour of his death, he had several other attacks—one very long and almost fatal—during which my son, M. W. Waterman, attended. Artificial respiration was resorted to with the same success.

CASE II.—Mr. E—— called upon me during the winter of 18—, to obtain a death certificate for a still-born child of seven months' gestation. I expressed a desire to see the child, and promised to visit him during the day. A midwife had assisted during the delivery. It was a cold stormy day, and 1 p. m. before I arrived at Mr. E——'s house. He lived in a low basement. Mr. E—— was a Hebrew, and according to Hebrew rites, the child had been laid upon a little straw upon the ground, and covered with a light black shawl. It had thus been lying since 5 a. m. As I was examining the child, I could detect some slight twitching movement over the region of the heart. I watched attentively, and observed the movement again. I had the child removed from the ground and placed upon a pillow on the table. The child was cold as ice but not rigid. I could detect no heart's sound, nor any respiratory murmur, but the muscular twitchings were very evident. I immersed the child in a hot bath, and initiated artificial respiration. Twenty minutes passed in this seemingly hopeless work. Then the child opened its eyes. A little more work and respiration began, laborious and interrupted at first, more normal by degrees. The heart's action came up in good style, and a human life was saved! The child thus saved is now one of the most accomplished violinists in this city.

The preceding cases are well calculated to rouse our most serious attention. How often has it been within reach of the physician thus to save life if but strict and critical performance of duty had been attended to! How often are certificates of death written out without first scrutinizing the body, and ascertaining, by all means at our command, whether death has really claimed his victim irrevocably!

Mr. B——'s case, especially, offers much encouragement to try revivification in sudden deaths, especially in heart disease. One case thus given back to life and light forms a recollection bright and pleasant upon the thorny path of a physician's life.—*N. Y. Med. Record.*

